# First Description of the Mitogenome Features of *Neofoleyellides* Genus (Nematoda: Onchocercidae) Isolated from a Wild Bird (*Pyrrhocorax pyrrhocorax*)

**DOI:** 10.3390/ani12202854

**Published:** 2022-10-20

**Authors:** Tingting Wu, Xiaoxiao Ma, Fengfeng Wang, Linhong Xie, Qingbo Lv, Minhao Zeng, Yu Xu, Siyuan Qin, Qiaocheng Chang

**Affiliations:** 1 School of Public Health, Shantou University, Shantou 515063, China; 2 College of Animal Science and Veterinary Medicine, Heilongjiang Bayi Agricultural University, Daqing 163319, China; 3 Center for Biological Disaster Prevention and Control, National Forestry and Grassland Administration, Shenyang 110034, China

**Keywords:** Onchocercidae nematodes, *Neofoleyellides* sp., corvid, mt genome, phylogenetic analyses

## Abstract

**Simple Summary:**

Filarioidea, a superfamily of nematodes, presently includes 42 species divided into six genera, mainly in the family Onchocercidae, which have been reported to infect a wide range of hosts, including reptiles, birds, and mammals. Current limitations in molecular characterization methods and species identification are the main obstacles to a better understanding of the biology of Onchocercidae species, particularly in wildlife. Thus, the objective of the present study was to sequence and analyze the complete mt genome of *Neofoleyellides* sp. isolated from a wild bird (*Pyrrhocorax pyrrhocorax*) and to assess its phylogenetic position in the Onchocercidae family. The evaluated *Neofoleyellides* sp. mt genome was consistent with the molecular pattern of the Onchocercidae family: 36 subunits consisting of 12 PCGs, 2 rRNAs, and 22 tRNAs. Phylogenetic analyses based on the 18S rRNA gene, cox1 gene, and 12 PCGs showed consistent results, which strongly supported monophyly of the genus *Neofoleyellides*. These findings enriched the gene database and improved our knowledge of the molecular characteristics of the Onchocercidae family, which provide useful genetic markers to study the population genetics, molecular biology, and phylogenetics of these Onchocercidae nematodes.

**Abstract:**

The Onchocercidae family is composed of more than 30 valid nematode species with notable zoonotic potential. Current limitations in molecular characterization methods and species identification are the main obstacles to a better understanding of the biology of Onchocercidae species, particularly in wildlife. This study describes for the first time the complete mitochondrial (mt) genome sequence of *Neofoleyellides* sp. isolated from a wild bird (*Pyrrhocorax pyrrhocorax*) and belonging to the *Neofoleyellides* genus (Nematoda: Onchocercidae). The mt genome of *Neofoleyellides* sp. (GenBank accession number: ON641583) was a typical circular DNA molecule of 13,628 bp in size with an AT content of 76.69%. The complete mt genome comprised 36 functional subunits, including 12 protein-coding genes (PCGs), 2 ribosomal RNA genes, and 22 transfer RNA genes. The most common start codon was ATT/ATG except for *nad*2 with TTG, and TAA was the termination codon for all protein-coding genes (PCGs). Phylogenetic analysis of the concatenated and aligned amino acid sequences of the 12 PCGs showed that the trees generated using different methods (Bayesian inference and maximum likelihood) with different partition schemes shared similar topologies. The isolated *Neofoleyellides* sp. was placed in the Onchocercidae family and formed a sister branch with the genera *Onchocerca* and *Dirofilaria*. The entire mt genome of *Neofoleyellides* sp. presented in this study could provide useful data for studying the population genetics and phylogenetic relationships of Onchocercidae species.

## 1. Introduction

Filarioidea, a superfamily of nematodes, presently includes 42 species divided into six genera, mainly in the family Onchocercidae, with a worldwide distribution [1]. Onchocercidae nematodes are transmitted by blood-sucking arthropods, and some genera such as *Dirofilaria* and *Onchocerca* have been linked to cases of blindness in humans [2]. Blood parasites have been the focus of various studies, and several reports have indicated that the microfilariae of nematodes of the Onchocercidae family can be found in the blood of birds [3,4]. Onchocercidae nematodes have been reported to infect a wide range of hosts, including reptiles, birds, and mammals [5,6]. Although some Adult worms of Onchocercidae have been detected in different tissues of bird hosts, there is limited information on wild birds [7]. Therefore, determining their presence in the final host is still challenging. Most of the previous research on *Neofoleyellides* has focused on the host of amphibians, and the phylogenetic relationships of amphibian onchocercids has been examined using molecular genetic methods (18S rDNA and *cox*1 gene sequences), in which all species parasitizing amphibians were recovered as the most distantly related genera of the family tree [5,8]. However, relationships between *Foleyellides*, *Neofoleyellides,* and *Ochoterenella* remained unresolved. In addition, there is limited phylogenetic information on adult *Neofoleyellides* worms in birds; additionally, the molecular detection of these parasites has not been performed.

*Pyrrhocorax pyrrhocorax*, a large corvid with a body length of 36–48 cm, mainly feeds on locusts, grasshoppers, mosquitoes and ants, which share a similar diet to amphibians; thus, it carries the same risk of *Neofoleyellides* infection. Unpredictable changes in ecological, biological, and social conditions caused by globalization have led to alterations in the prevalence, spread, and geographical range of infections. Therefore, the continuous surveillance of emerging infectious diseases is crucial, especially for emerging zoonotic parasites such as Onchocercidae nematodes, whose species diversity remains insufficiently explored [9].

Although the morphological and biological aspects of the parasite species were targeted for a phylogenetic reconstruction, the rapid and accurate identification of vector species is difficult due to the morphological homogeneity between species at certain life stages and the lack of necessary taxonomic expertise [10]. Molecular methods are of great significance for simplifying the detection of filarioids in hosts and species identification [2]. The strict maternal inheritance of the mitochondrial (mt) genome, lack of recombination, and rapid evolutionary rates have been widely used to determine species evolution, especially for classification at high taxonomic levels [11]. Furthermore, phylogenetic analysis based on molecular methods can be used to identify sister species (closest relatives in the phylogenetic tree) that share a common ancestor with the species of interest, and is useful to understand their role in the transmission cycle of pathogens [12]. 

The limited molecular characterization information of the avian Filarioidea species remains a major obstacle to a better understanding of the biology of these parasites, especially in wild animals. Thus, the objective of the present study was to sequence and analyze the complete mt genome of *Neofoleyellides* sp. isolated from a wild bird (*Pyrrhocorax pyrrhocorax*) and to assess its phylogenetic position in the Onchocercidae family. The findings could provide novel and useful genetic markers for further studies on species identification and molecular epidemiology.

## 2. Materials and Methods

### 2.1. Sample Collection, Total DNA Extraction and Molecular Detection

A total of three Adult *Neofoleyellides* worms were isolated from the intestinal tract of the wild bird *Pyrrhocorax pyrrhocorax* in Xining City, Qinghai Province, China (36°36′ N, 101°77′ E). The collected specimens were identified based on external morphological characteristics using dichotomous keys to represent the Filarioidea superfamily [9]. The collected worms were thoroughly washed with physiological saline three times and fixed in 75% (*v*/*v*) ethanol at −80 °C until use.

In order to identify the worm, total genomic DNA was extracted from all three adult worms using the TIANamp Genomic DNA Kit (Tiangen, Beijing, China) according to the manufacturer’s instructions. Molecular identification was conducted by polymerase chain reaction (PCR) targeting the 18S ribosomal RNA (rRNA) and mitochondrial *cox*1 gene, which has been previously described in several studies [9,13]. The primers are listed in Table S1. During amplification of both gene sequences, PCR was performed in the same reaction system: a 25 μL system including 12.5 μL of 2× Ex Taq Mix PCR buffer (Takara), 0.5 μm of each primer, 1.5 μL of DNA sample, and 10 μL of nuclease-free water. 

### 2.2. Construction of the Genomic Library and Sequencing

For the genomic library construction, Total DNA was extracted from one adult worm, and was standardized at a concentration of 0.3 ng/L. The Illumina NovaSeq sequencing platform was used to adopt the whole-genome shotgun (WGS) strategy based on next-generation sequencing. After sequencing, a DNA library was constructed with an insert size of 400 bp using the paired-end method, which was conducted by Personalbio (Nanjing, China). The sequenced raw data were transferred to a computer workstation, and the analysis and genomic characterization steps were performed. The quality metrics of the obtained data were assessed using FastQC v. 0.11.9 software (available online: https://www.bioinformatics.babraham.ac.uk/projects/fastqc/ (accessed on 1 August 2019)), and Trim Galore v. 0.6.5 was used to remove adapter sequences (available online: https://www.bioinformatics.babraham.ac.uk/projects/trim_galore/ (accessed on 19 November 2019)). Finally, further quality checks and data validation were performed using FastQC.

### 2.3. Genomic Assembly

The A5 miseq v20150522 and SPAdes v3.9.0 softwares were used to construct contig and scaffold sequences, respectively, which were examined using pre-processed high-quality second-generation sequencing data for each species [14,15]. Furthermore, collinearity analysis was conducted using the Mummer v3.1 software to fill gaps among the contigs [16], and the corrected mt sequence was obtained using the Pilon v1.18 software [17]. Finally, the assembled mt genome sequence was compared with reference Onchocercidae family sequences (*Onchocerca lupi*, GenBank ID: NC056960; *Wuchereria bancrofti*, GenBank ID: AP017705) and manually validated. 

### 2.4. Annotation and Bioinformatic Analysis

The complete mt genome sequence was functionally annotated using the MITOS web server (http://mitos.bioinf.uni-leipzig.de/index.py (accessed on 16 June 2017)) [18], and the secondary structures of transfer RNAs (tRNAs) were identified using tRNAscan-SE (http://lowelab.ucsc.edu/tRNAscan-SE (accessed on 4 May 2021)). An online open reading frame finder (https://www.ncbi.nlm.nih.gov/orffinder/ (accessed on 4 May 2021)) was used to analyze and translate protein-coding genes (PCGs).

DNAStar (v. 5.0) was used to calculate the A+T and G+C contents of each gene, and the AT skew and GC skew values were calculated using the formulas AT skew = (A − T)/(A + T) and GC skew = (G − C)/(G + C) for the coding genes [19]. Synonymous codon usage bias in PCGs was analyzed using CodonW [20], and relative synonymous codon usage (RSCU) values were determined. Sliding window analysis and pairwise comparisons of the proportions of non-synonymous (*dN*) and synonymous (*dS*) substitutions (*dN/dS*) among the obtained sequences were performed using DnaSP (v. 6) [21]. Sliding window analysis was used to estimate the nucleotide diversity (π) for every 200 bp (in 25 bp overlapping steps), and nucleotide diversity was plotted according to the midpoint position.

### 2.5. Phylogenetic Analysis

The concatenated amino acid sequences of the complete *Neofoleyellides* sp. mt genome were aligned with the corresponding amino acid sequences of 12 Filarioidea species available in GenBank, using *Gongylonema pulchrum* (NC026687) as outgroup. All 12 PCG sequences obtained in this study were extracted together with those of other taxa available in the GenBank data repository, and the MAFFT software with the option (L-INS-I) was also employed to determine the gene boundaries [22]. Phylogenetic relationships between the analyzed species were determined using two methods: maximum likelihood (ML) and Bayesian inference (BI). MEGA X software was used for the ML method, and bootstrapping was performed with 1000 replicates. MrBayes 3.1 was used to construct the BI tree, and four independent Markov chain runs were performed for 1,000,000 metropolis-coupled MCMC generations, sampling a tree every 100 generations. The first 25% (2500) of the trees were omitted as burn-ins, and the remaining trees were used to calculate Bayesian posterior probabilities. Phylogenetic trees were visualized using FigTree (v. 1.42) [23].

## 3. Results and Discussion

### 3.1. Acquisition of 18S rDNA and Mitochondrial cox1 Genes

The specimen was identified as *Neofoleyellides* sp., confirmed by PCR-based sequencing of 18S rDNA and *cox*1 genes. The amplified 18S rDNA showed 96.9% of nucleotide identity with that of *Neofoleyellides* spp. (GenBank accession number: MW599275). Besides, the *cox*1 gene was identical for 98.67% to the sequence of *Neofoleyellides* sp. (GenBank accession number: MW774895). Phylogenetic analyses of the 18S rDNA and *cox*1 gene were placed in the same genus of *Neofoleyellides* (Figures S1 and S2). Filarial nematodes are widespread parasites that infect all classes of vertebrates except fish. Many of these are of socioeconomic and medical importance [1]. To date, only a few studies on the *Neofoleyellides* genus have focused on the phylogenetic relationships of amphibian onchocercids based on 18S rDNA and *cox*1 gene sequences, and there is a lack of information for birds [24]. Birds play an important role in the spread of diseases, and a timely and accurate identification of the pathogens they carry is particularly important for the prevention and control of unknown diseases. In this study, we reported for the first time the mitochondrial genome of *Neofoleyellides* sp. isolated from a wild bird *Pyrrhocorax pyrrhocorax*, which suggests that *Neofoleyellides* are not only parasitic on amphibians, but may have a wider host distribution.

### 3.2. Mitogenome Organization and Composition

**Figure 1:**
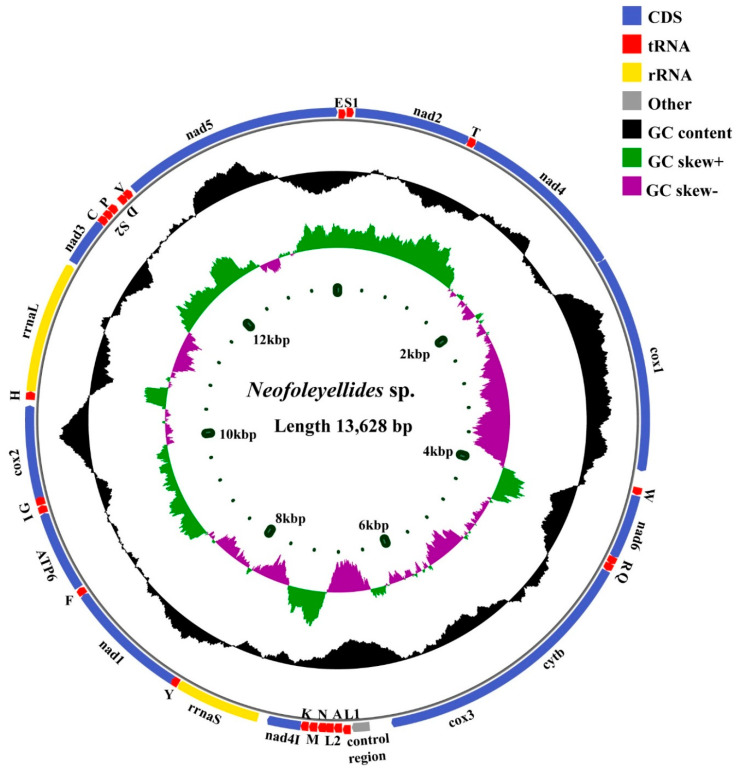
Structural representation of *Neofoleyellides* sp. mitogenome. From inside to outside, the first circle represents the length scale, the second circle represents the GC skew, the third circle represents the GC content, and the fourth circle represents the arrangement of PCGs, tRNAs, and rRNAs; each tRNA is identified by a unique letter abbreviation.

The complete mt genome of *Neofoleyellides* sp. (GenBank accession number: ON641583) was a typical circular DNA molecule, 13,628 bp in size and contained 36 functional and conserved subunits: 12 PCGs (including *cox*1-3, *nad*1-6, *nad*4L, *atp*6, and *cyt*b), 22 tRNAs, and 2 rRNAs (*rrn*L and *rrn*S) (Figure 1). Similar to the genes of other Onchocercidae nematodes, all genes were organized along the N (forward) strands (Table 1). Taking into account the PCGs, tRNAs, and rRNAs, the mitogenome had an average AT content of 76.69%, which was similar to that of previously reported mt genomes including those of *D. repens* (76.06%), and *B. malayi* (75.46%) available in GenBank (NC029975, NC004298).

**Figure 2:**
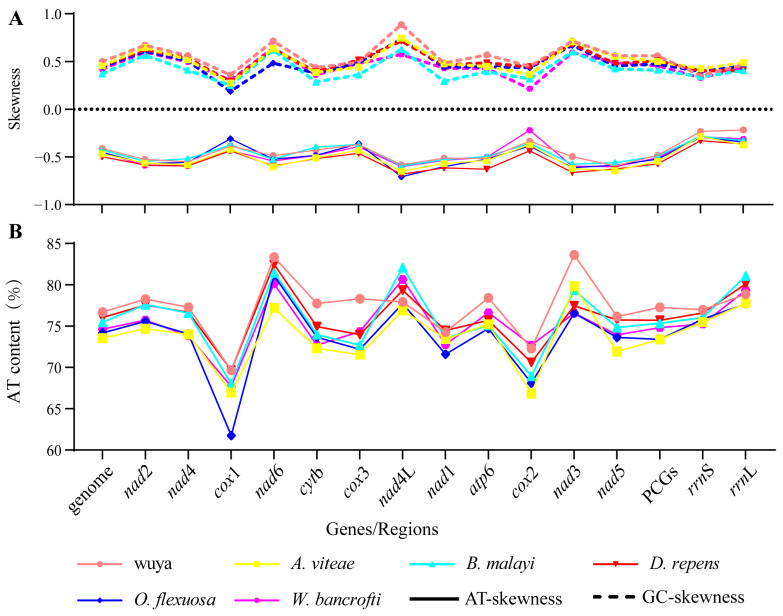
AT/GC contents and AT/GC skews of the investigated mitogenomes. (**A**) AT GC skews. (**B**) AT contents (%).

Among the 12 PCGs, *nad*3 had the highest A+T content (83.62%), followed by *nad*6 (83.33%), and *atp*6 (78.41%). Although *cox*1 had the lowest A+T content, it was still greater than 69.65%. A high AT content indicates a greater proportion of adenine and cytosine than thymine and guanine on the majority chain [25]. Analysis of the compositional asymmetry of the total mitogenome yielded negative values for AT skews (−0.41) and positive values for GC skews (0.50), which are consistent with the values for nematode parasites [26]. The AT skew values within the mt genome ranged from −0.22 (*rrn*L) to −0.59 (*nad*5), and the GC skew values ranged from 0.35 (*rrn*S) to 0.89 (*nad*4L) (Figure 2). The control region, which is known to initiate replication in vertebrates and invertebrates, was located between *cox*3 and *trn*A and varied in size. The control region (also known as the AT-rich region) is naturally rich in homopolymers and highly variable in terms of the length and rates of mutations, which may constitute an impairment to next-generation sequencing [27,28]. Alternative methods can be used to address this shortcoming; conventional PCR and Sanger sequencing allow target amplification, as demonstrated in similar studies [29]. 

The locations of the two rRNA genes (*rrn*S and *rrn*L) were identical in the mt genome, where *rrn*S was flanked by *nad*4L and *trn*Y, and *rrn*L was flanked by *trn*H and *nad*3; the locations of these rRNA genes are similar to those in other filarial parasites [30]. The mt genome is unable to express all of the components required to assemble mt protein complexes. tRNAs play an essential role in amino acid transport, and the remaining proteins and RNAs involved in mt biogenesis are transported from the cytoplasm to the mitochondria [31]. The total length of the 22 tRNAs was 1222 bp, and the individual gene lengths varied from 51 bp to 60 bp (Table 1). The most common nucleotide mismatch was G-U, followed by U-U, which play an important role in maintaining the stability of the tRNA secondary structure [32].

### 3.3. Characteristics of PCGs

The length of the PCG region in *Neofoleyellides* sp. was 10,358 bp, and the AT content was 77.27%. The relative lengths of PCGs were in the following order: *cox*1 > *nad*5 > *nad*4 > *cyt*b > *nad*1 > *nad*2 > *cox*3 > *cox*2 > *atp*6 > *nad*6 > *nad*3 > *nad*4L. All 12 PCGs (except *nad*2 with TTG) had ATN as the start codon, with ATT being the most common (*nad*6, *nad*4L, *cox*2, *nad*4, *nad*4L, *nad*3, and *nad*5), followed by ATG (*cox*1, *cyt*b, *nad*1, and *nad*6), all of which had TAA as the standard termination codon (Table 1). However, in most Onchocercidae family species, *nad*2 uses TTG as the start codon, which is considered a common feature across various organisms [33]. Termination codons for almost all PCGs are typically complete and incomplete stop codons (T or TA), which have also been observed in all metazoan mitogenomes and can be converted to the complete termination codon TAA through post-transcriptional modification [32].

**Figure 3:**
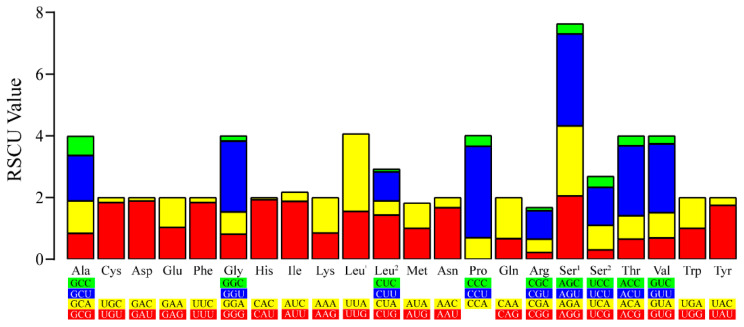
Relative synonymous codon usage (RSCU) values of the obtained mitogenomes. RSCU values are represented on the y-axis, and codons for the amino acids of the Onchocercidae family are represented on the x-axis.

The PCGs in the mt genome of *Neofoleyellides* sp. contained 3479 amino acids, including 176 strongly basic (**+**) amino acids (K, R), 156 strongly acidic (-) amino acids (D, E), 1852 hydrophobic amino acids (A, I, L, F, W, V), and 800 polar amino acids (N, C, Q, S, T, Y). RSCU analysis showed that nearly all codons were used in the mt genome, except for CCG (Pro). CCU (Pro) was the most common codon in the *Neofoleyellides* sp. mt genome with an RSCU value of 2.52, followed by UUA (Leu^1^) with an RSCU value of 2.23. Except for CCG (Pro), CAC (His) was the least common codon in the mt genome with an RSCU value of 0.07 (Figure 3). Synonymous codon usage bias may be attributed to various factors including gene function, recombination, mutation bias, GC composition, gene length, codon position, environmental stress, and population size [34]. Codons ending with A or U were more common than those ending with CG or GC, which is a common feature of some species [35].

**Figure 4:**
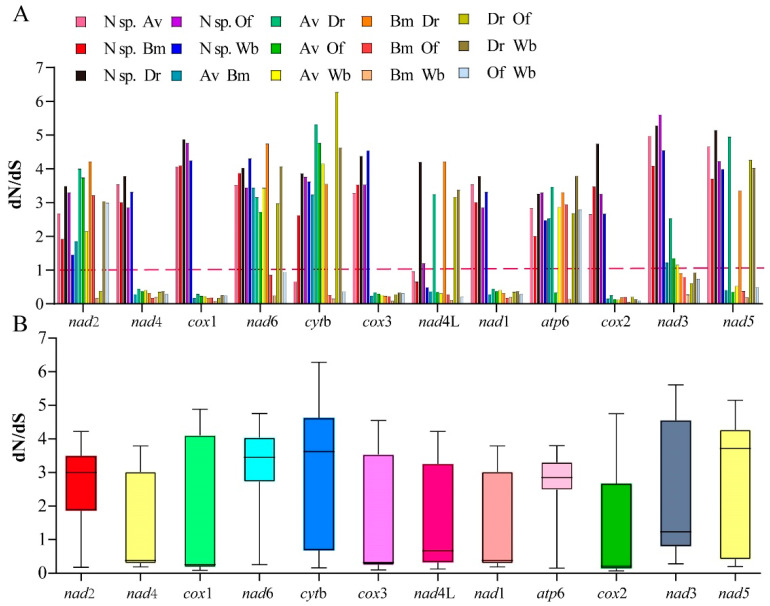
Ratio of the rates of non-synonymous (*dN*) to synonymous (*dS*) nucleotide substitutions (*dN/dS*). (**A**) Bar graph of the pairwise ratios of *dN/dS* for each of the mitochondrial subunits of the investigated species. (**B**) Box chart illustrating the average pairwise ratios of *dN/dS* for each of the mitochondrial subunits of the investigated species. The *dN/dS* ratios are plotted on the y-axis, and the PCGs are plotted on the x-axis.

**Figure 5:**
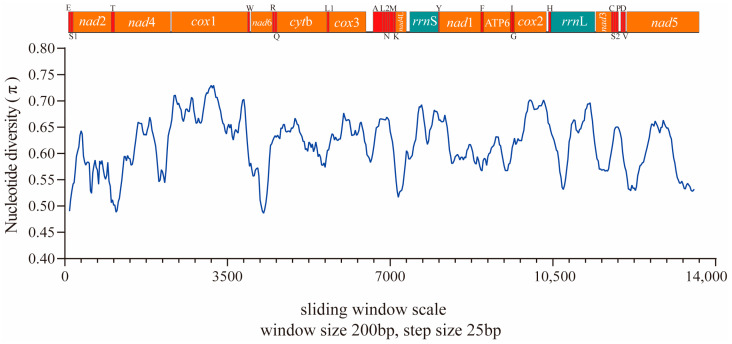
Nucleotide diversity (π) of the mitogenomes obtained in this study. The blue line indicate nucleotide diversity values based on the evaluation of sequences from *B. malayi*, *D. repens*, *O. flexuosa*, and *A. viteae*. The π values (plotted on the y-axis) were calculated in a 200 bp sliding window with 25 bp steps. The lengths of the aligned sequences of the two evaluated groups are plotted on the x-axis. The limits of each gene are indicated above the graph. Vertical red bars represent tRNAs, orange rectangles represent PCGs, and cyan bars represent *rrn*L and *rrn*S.

To investigate the evolutionary pressure acting on different regions of the protein-coding mt genome, the corresponding sequences of each PCG in the studied species were paired based on the non-synonymous and synonymous substitution (*dN/dS*) ratio. The results indicated that different regions have evolved globally under the effect of negative (or purifying) pressure. The order of influence of evolutionary pressure according to the averages obtained was as follows: *cox*2 < *cox*1 < *nad*1 < *cox*3 < *nad*4 < *nad*4L < *nad*3 < *atp*6 < *nad*2 < *nad*6 < *cyt*b < *nad*5 (Figure 4). The *dN/dS* ratio is a measure of the selective pressures acting on genes, indicating neutral selection (*dN/dS* = 1), negative or purifying selection (*dN/dS* < 1), and positive or diversifying selection (*dN/dS* > 1) [36,37]. Previous studies have reported that the *dN/dS* ratios of four protein-coding genes (*nad*2, *nad*3, *nad*5, and *nad*6) are > 1, suggesting that these genes have evolved under positive or diversifying selection [26]. However, the *dN/dS* ratio range in our study was large because of the lack of complete mitogenome data for the *Neofoleyellides* genus. In addition to *dN/dS* analysis, nucleotide diversity was compared between the mt sequences obtained in the present study and those of other members of the Onchocercidae family. Nucleotide diversity was calculated for a 200 bp window, plotted against the midpoint of this window, and moved in 25 bp steps across the alignment. The values of nucleotide diversity (π) of the evaluated sequences were in the range of 0.48–0.73 (Figure 5). 

### 3.4. Phylogenetic Analyses

**Figure 6:**
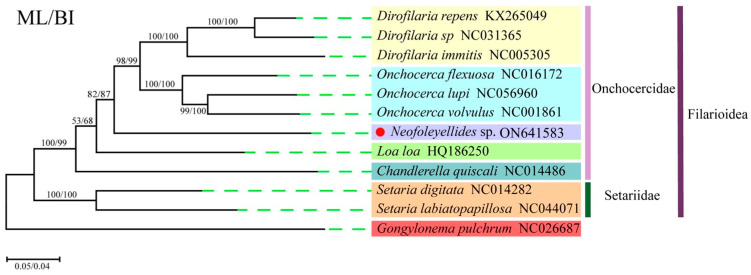
Phylogenetic reconstruction based on the concatenated amino acid sequences of 12 PCGs of 12 mitogenomes analyzed using the maximum likelihood and Bayesian inference methods. The sequenced specie of *Neofoleyellides* sp. in this study are marked with a red circle.

Phylogenetic analysis of the protein-coding regions was performed with 12 PCGs from 12 taxa (1 from this study and 11 from the GenBank database) using two analytical approaches (BI and ML) with *G. pulchrum* (Diptera: Spiruroidea) as the outgroup (Figure 6). In general, trees generated using different methods with different partition schemes share similar topologies. The Filarioidea superfamily had a topology with two well-supported clades corresponding to the families Setariidae and Onchocercidae. The Onchocercidae clade, which contained nine species, had five subclades representing the genera *Dirofilaria*, *Onchocerca*, *Neofoleyellides*, *Loa*, and *Chandlerella*, which is consistent with the results of previous studies [26,38]. In this study, the isolated *Neofoleyellides* sp. was placed in the Onchocercidae family, which formed a sister branch with the genera *Onchocerca* and *Dirofilaria* (posterior probability = 100). However, due to the lack of mt genome data for the *Neofoleyellides* genus, the adult worm formed an independent branch in the Onchocercidae family. Nevertheless, combined with 18S and *cox*1 gene evolution results, we determined that the parasites isolated from *Pyrrhocorax pyrrhocorax* belonged to the *Neofoleyellides* genus.

In previous studies, morphological and biological aspects were targeted for phylogenetic reconstruction; however, this method has drawbacks, especially in cases with diverse and ancient taxa [39]. Various studies have demonstrated that mtDNA sequences are valuable genetic markers for phylogenetic studies of members of Nematoda [39,40], and the advent of molecular tools has allowed a deeper investigation of the taxonomic relationships between organisms [41]. To date, only a few species have been confirmed to be infected by *Neofoleyellides* parasites, and more data are needed to understand their diversity and distribution, especially in wild animals [9]. However, the effect of the species of *Neofoleyellides* on host health has not been specifically investigated; only some pathological changes have been observed in frogs [24]. In addition, there is limited phylogenetic information on birds infected with *Neofoleyellides* parasites; thus, whether these parasites contribute to a risk of zoonosis is also worthy of further investigation. Therefore, mt characterization studies and the consequent inclusion of more filarial nematodes in public databases would allow more comprehensive phylogenetic reconstruction analyses and aid the elucidation of taxonomic relationships.

## 4. Conclusions

The present study is the first to determine the complete mt genome sequences of *Neofoleyellides* sp. isolated from a wild bird *Pyrrhocorax pyrrhocorax*. The evaluated *Neofoleyellides* sp. mt genome was consistent with the molecular pattern of the Onchocercidae family: 36 subunits consisting of 12 PCGs, 2 rRNAs, and 22 tRNAs. Phylogenetic analyses based on the 18S rRNA gene, *cox*1 gene, and 12 PCGs showed consistent results, which strongly supported monophyly of the genus *Neofoleyellides*. These findings enriched the gene database and improved our knowledge on the molecular characteristics of the Onchocercidae family, which provides useful genetic markers to study the population genetics, molecular biology, and phylogenetics of these Onchocercidae nematodes.

## Figures and Tables

**Table 1:** Mitochondrial genome organization of *Neofoleyellides* sp.

Genes	Strand	Position and Length (bp)	Initiation Codon	Stop Codon	Anticodon
*trn*E	N	1–57 (57)			TTC
*trn*S1	N	58–108 (51)			TCT
*nad*2	N	108–944 (838)	TTG	TA(A)	
*trn*T	N	944–999 (56)			TGT
*nad*4	N	1001–2224 (1224)	ATA	TAA	
*cox*1	N	2226–3878 (1653)	ATG	TAG	
*trn*W	N	3879–3933 (55)			TCA
*nad*6	N	3939–4400 (462)	ATT	TAA	
*trn*R	N	4399–4452 (54)			ACG
*trn*Q	N	4452–4505 (54)			TTG
*cyt*b	N	4509–5592 (1084)	ATG	T(AA)	
*trn*L1	N	5592–5647 (56)			TAG
*cox*3	N	5644–6426 (783)	ATA	TAA	
*trn*A	N	6777–6833 (57)			TGC
*trn*L2	N	6836–6895 (60)			TAA
*trn*N	N	6890–6946 (57)			GTT
*trn*M	N	6951–7009 (59)			CAT
*trn*K	N	7013–7068 (56)			CTT
*nad*4L	N	7069–7308 (240)	ATT	TAA	
*rrn*S	N	7310–7985 (676)	ATA	TAA	
*trn*Y	N	7986–8039 (54)			GTA
*nad*1	N	8042–8911 (870)	TTG	TAG	
*trn*F	N	8911–8964 (54)			GAA
*atp*6	N	8971–9549 (579)	ATG	TAA	
*trn*I	N	9553–9607 (55)			GAT
*trn*G	N	9612–9669 (58)			TCC
*cox*2	N	9669–10,358 (690)	ATT	TAG	
*trn*H	N	10,358–10,416 (59)			GTG
*rrn*L	N	10,418–11,386 (969)	ATT	T(AA)	
*nad*3	N	11,380–11,721 (342)	ATT	TAA	
*trn*C	N	11,720–11,774(55)			GCA
*trn*S2	N	11,776–11,826(51)			TGA
*trn*P	N	11,828–11,882 (55)			AGG
*trn*D	N	11,922–11,976 (55)			GTC
*trn*V	N	11,976–12,029 (54)			TAC
*nad*5	N	12,029–13,621 (1593)	ATT	TAA	

## Data Availability

All data generated during this study are available as tables and figures included in this published article and its supplementary information files. The genome sequence data that support the findings of this study are available in GenBank of NCBI at (https://www.ncbi.nlm.nih.gov/ accessed on 18 October 2022) under the accession no. ON641583.

## Supplementary Materials

The following supporting information can be downloaded at: https://www.mdpi.com/article/10.3390/ani12202854/s1. Figure S1. Phylogenetic reconstruction based on the 18S rRNA gene using the maximum likelihood method with *Setaria yehi* as the outgroup. The sequenced 18S gene of *Neofoleyellides* sp. was marked with a red circle. Figure S2. Phylogenetic reconstruction based on the cox1 gene using the maximum likelihood method with Setaria yehi as the outgroup. The sequenced cox1 gene of *Neofoleyellides* sp. was marked with a red circle. Table S1. Polymerase chain reaction primers used to verify the 18S and *cox*1 genes of *Neofoleyellides* sp.
