# Supplementary material for: First Description of the Mitogenome Features of Neofoleyellides Genus (Nematoda: Onchocercidae) Isolated from a Wild Bird (Pyrrhocorax pyrrhocorax)
Source: Animals (Basel). 2022 Oct 20;12(20):2854. doi: 10.3390/ani12202854 (PMC9597759; doi:10.3390/ani12202854)
Supplement: Supplementary file 1 [file animals-12-02854-s001.zip › animals-1979756-supplementary.pdf]

Table S1. Polymerase chain reaction primers used to verify the 18S and *cox*1 genes of *Neofoleyellides* sp.

Gene	Primer	5'-3'
18S	18SF	5′-GAC GGG CAG CTT CCG GAA ACG-3
	18SR	5-CCG CTT TTC TCG AAA CGG CTCA-3′
COXI	COI-intF	5′-TGA TTG GTG GTT TTG GTA A-3′
	COI-intR	5′-ATA AGT ACG AGT ATC AAT ATC-3′

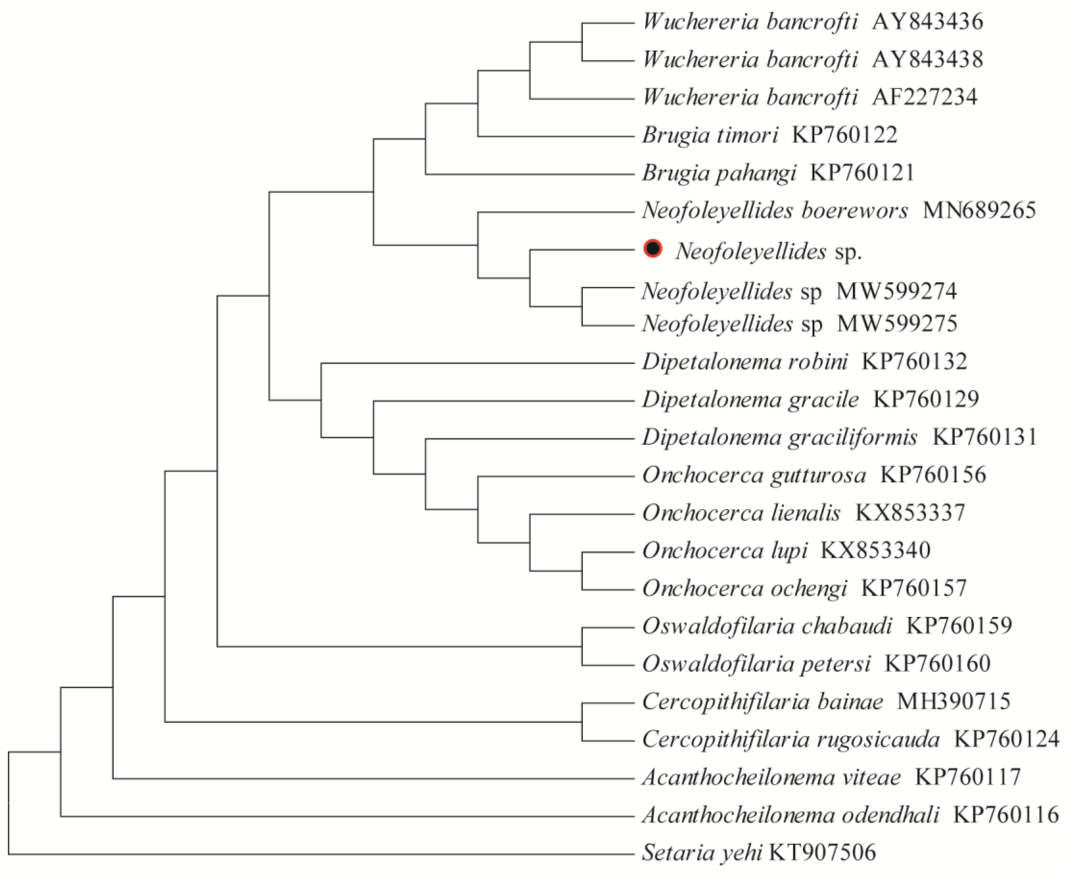

Figure S1. Phylogenetic reconstruction based on the 18S rRNA gene using the maximum likelihood method with *Setaria yehi* as the outgroup. The species sequenced in this study are marked with a red circle.

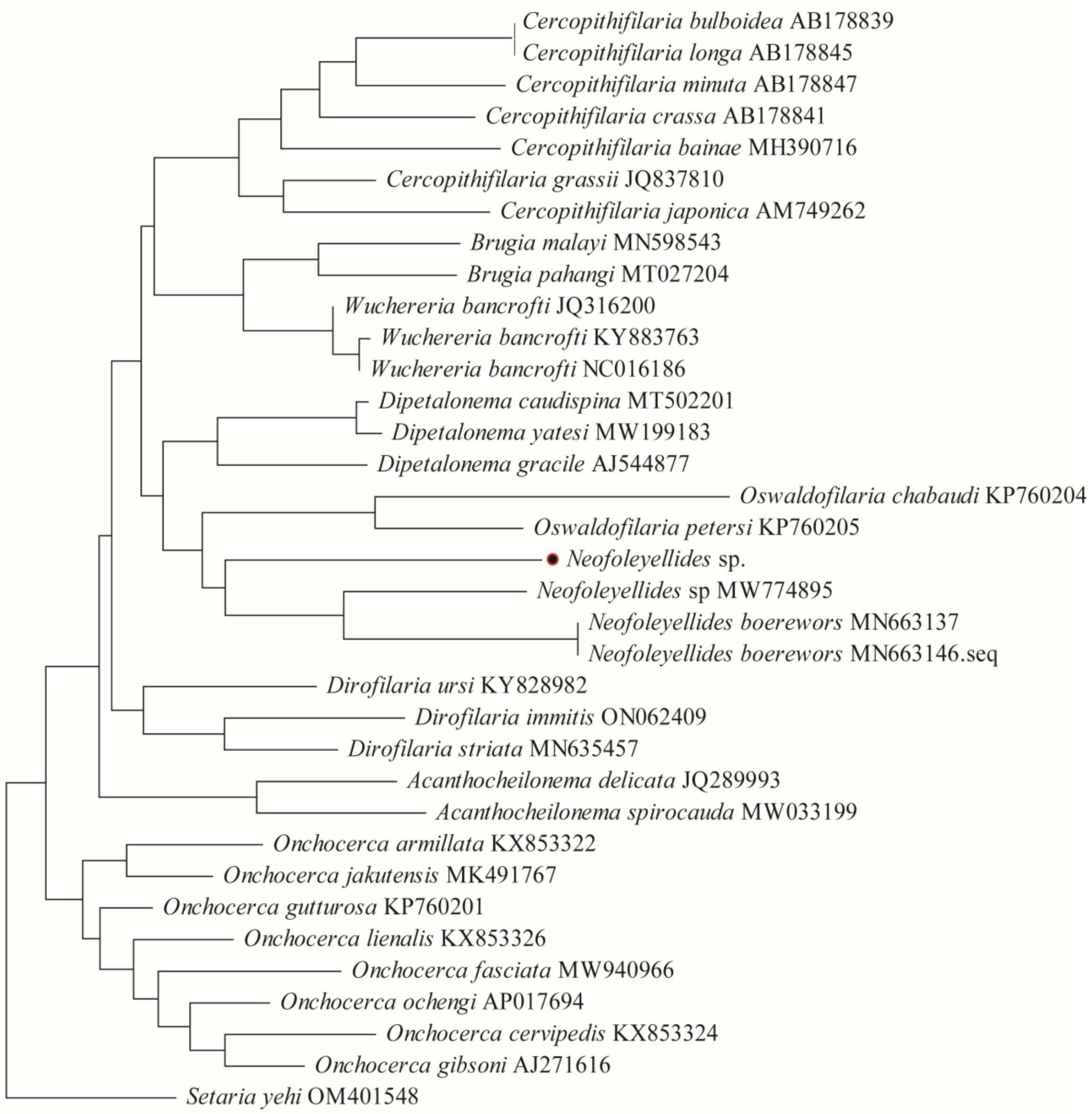

Figure S2. Phylogenetic reconstruction based on the *cox*1 gene using the maximum likelihood method with *Setaria yehi* as the outgroup. The species sequenced in this study are marked with a red circle.
